# The Underlying Pathology of Atherosclerosis: Different Players

**DOI:** 10.3390/ijms23063235

**Published:** 2022-03-17

**Authors:** Noemi Rotllan

**Affiliations:** 1 Institut d’Investigació Biomèdica IIB Sant Pau (IIB SANT PAU), 08041 Barcelona, Spain; nrotllanv@santpau.cat; 2 CIBER de Diabetes y Enfermedades Metabólicas Asociadas (CIBERDEM), 28029 Madrid, Spain

Cardiovascular diseases (CDVs) are still the leading cause of mortality in the developed world, despite the high number of deaths caused by the COVID pandemic of the last two years. According to the World Health Organization (WHO), CDVs are the cause of 32% of all global deaths. Atherosclerosis is the primary cause of CDVs. Several aspects contribute to the formation of plaque: lipid metabolism dysregulation; unbalanced cholesterol levels in plasma; and the dysfunction and inflammation of the three key components of the arterial wall—the endothelial cells, vascular smooth muscle cells, and macrophages. Large amounts of effort and resources have been dedicated to deciphering the different players that control the molecular mechanisms that lead to the formation of plaque. Interestingly, non-coding RNA has emerged as an important transcriptional and post-transcriptional regulator of target genes during the development of atherosclerosis. Moreover, in the era of precision nanomedicine, much effort has devoted to the development of therapeutic tools capable of specific direct treatments for those key cells that play an important role during the progression of atheroma. I am pleased to introduce this Special Issue, “Molecular Mechanism and Pathophysiology of Atherosclerosis”, the aim of which is to present a wide view of the different players that are critical in the pathophysiology of atherosclerosis.

The dysregulation of cholesterol metabolism in macrophages is one of the primary and most important processes related to the pathophysiology of atherosclerosis, which is controlled by different key players. In the extensive review by Javadifar et al., the authors discuss the role of microRNAs as the therapeutic target of genes that are involved in the uptake, esterification, and efflux of cholesterol, which are the main steps in the formation of foam cells. The authors also point out the limitations of using inhibitors of miRNAs, which should be taken into consideration, as well as the off-target effects of these therapies [1].

Discovered in the late seventies, the classic drugs used to treat dyslipidemias are statins. They are still widely used, although other drugs for reducing LDL-C levels, such as ezetimibe or PCSK9 inhibitors, have appeared more recently. However, the risk of developing atherosclerotic cardiovascular disorders remains high. Other possible therapies for the treatment of patients with hypercholesterolemia who show little response to traditional therapies or have no LDLR activity have also been studied, such as ANGPTL3. In their review, Pei-Yi Chen et al. list the different strategies for the pharmacological inactivation of ANGPTL3: the use of blocking monoclonal antibodies such as evinacumab; antisense oligonucleotides, such as vupanorsen; or the use of CRISPR genome editing systems, such as base editor 3 (BE3). The authors point out that, to date, the absence of oral and affordable low-molecular-weight ANGPTL3 inhibitors has limited their beneficial effect, and more research therefore needs to be done [2].

The dysfunction and inflammation of the different cells of the vascular wall are also key players in the formation of atherosclerotic plaque. Demyanets et al. describe the paradigm changes in interleukin (IL)-33 (IL-33) in vascular biology in their extensive review published in this Special Issue. They summarize recent data on the biology, structure, and signaling of this fascinating dual-function cytokine, which can regulate transcription or act as an extracellular cytokine. IL-33 may be the Dr Jekyll and Mr Hyde cytokine! In experimental models of vascular pathologies, it has been shown to have protective or harmful effects, depending on the conditions of the experiment, or the type or dose administered. Thus, many questions need to be addressed before a possible therapeutic modulation of IL-33 can be considered as a treatment for the formation of atherosclerotic plaque [3].

In another original article, González-Guerra et al. focus their attention on the interplay between blood pressure (BP) and the development of atherosclerosis. The authors hypothesize that persistent elevated BP increases plaque formation. In order to evaluate this causal link, they used a new mice model, with persistent high BP provoked by adeno-associated virus (AAV) gene transfer, specifically the *hRenin* and *hAngiotensinogen* genes. These mice had sustained systolic BP and large atherosclerotic lesions. Moreover, when BP was controlled with a calcium channel blocker, the development of atheroma plaques was attenuated. The authors suggest that earlier intervention to lower BP may prevent or delay the mortality and morbidity associated with atherosclerosis [4].

It is known that nutrients can modulate the expression of miRNAs at the cellular level and therefore have an impact on cellular physiology. For instance, betaine, choline, and L-carnitine nutrients found in animal products are metabolized into trimethylamine-n-oxide (TMAO), which has been associated with CVD risk. Díez-Ricote et al. evaluate how the effect of TMAO in macrophages and hepatocytes up-regulates the expression of miR-30c-5p and miR-21-5p [5]. Specifically, their results suggest that miR-30c affects the expression of *PER2*, which is a target gene that regulates the circadian system and has been linked to metabolic syndrome. Pre-clinical assays are needed to corroborate this. In another stimulating article, Pei-Yi Chen et al. demonstrate in cell lines that tangeretin (4′,5,5,7,8-pentamethoxyflavone), a dietary bioactive polymethoxyflavone found in citrus peel, inhibits the gene expression of *ANGPTL3* by counteracting the activity of LXRα [6]. The authors suggest that the mechanical inhibition of *ANGPTL3* by tangeretin may augment LPL catalytic activity, therefore resulting in a reduction in triglycerides in plasma. However, future experiments using animal models are required to support the idea that tangeretin may be useful as a therapeutic agent for dyslipidemia.

I hope that these articles and reviews, contributed by experts in this field, will provide valuable new data and resources to researchers studying atherosclerosis and lead to the development of innovative therapeutic tools.

## Data Availability

Not applicable.

## Abbreviations

HDL	High-density lipoprotein cholesterol
CVD	Cardiovascular disease
Apo	Apolipoprotein
CKD	Chronic kidney disease
LDL	Low-density lipoprotein
SAA	Serum amyloid A
EL	Endothelial lipase
PON	Paraoxonase

## References

[1] Javadifar A., Rastgoo S., Banach M., Jamialahmadi T., Johnston T.P., Sahebkar A. (2021). Foam Cells as Therapeutic Targets in Atherosclerosis with a Focus on the Regulatory Roles of Non-Coding RNAs. Int. J. Mol. Sci..

[2] Chen P.Y., Gao W.Y., Liou J.W., Lin C.Y., Wu M.J., Yen J.H. (2021). Angiopoietin-Like Protein 3 (ANGPTL3) Modulates Lipoprotein Metabolism and Dyslipidemia. Int. J. Mol. Sci..

[3] Demyanets S., Stojkovic S., Huber K., Wojta J. (2021). The Paradigm Change of IL-33 in Vascular Biology. Int. J. Mol. Sci..

[4] Gonzalez-Guerra A., Roche-Molina M., García-Quintáns N., Sánchez-Ramos C., Martín-Pérez D., Lytvyn M., de Nicolás-Hernández J., Rivera-Torres J., Arroyo D.F., Sanz-Rosa D. (2021). Sustained Elevated Blood Pressure Accelerates Atherosclerosis Development in a Preclinical Model of Disease. Int. J. Mol. Sci..

[5] Díez-Ricote L., Ruiz-Valderrey P., Micó V., Blanco-Rojo R., Tomé-Carneiro J., Dávalos A., Ordovás J.M., Daimiel L. (2021). Trimethylamine n-Oxide (TMAO) Modulates the Expression of Cardiovascular Disease-Related microRNAs and Their Targets. Int. J. Mol. Sci..

[6] Chen P.-Y., Chao T.-Y., Hsu H.-J., Wang C.-Y., Lin C.-Y., Gao W.-Y., Wu M.-J., Yen J.-H. (2021). The Lipid-Modulating Effect of Tangeretin on the Inhibition of Angiopoietin-like 3 (ANGPTL3) Gene Expression through Regulation of LXRalpha Activation in Hepatic Cells. Int. J. Mol. Sci..

